# The splice of life: an isoform-centric view of disease, technology, and therapeutics

**DOI:** 10.1172/JCI207476

**Published:** 2026-07-15

**Authors:** Timothy Pan, Lina Lu, Ruli Gao

**Affiliations:** 1 Department of Biochemistry and Molecular Genetics;; 2 Center for Cancer Genomics, Robert H. Lurie Cancer Center; and; 3 The Driskill Graduate Program, Northwestern University Feinberg School of Medicine, Chicago, Illinois, USA.

## Abstract

Alternative splicing is a pervasive mechanism that expands the coding potential and functional complexity of the human genome. Dysregulated isoform usage alters gene functions and contributes broadly to human disease across developmental, neurodegenerative, and cancer settings. Technologies for characterizing splicing and isoforms have advanced rapidly, evolving from Sanger sequencing of individual cDNA clones to high-throughput next-generation sequencing of splice junctions, and more recently to long-read sequencing that resolves full-length transcripts at bulk, single-cell, and spatial resolutions. With the growing recognition of their critical roles in human disease, multiple therapeutic modalities have been developed to precisely target splicing and isoform regulation at the DNA, RNA, and protein levels. Clinical-grade small molecules and antisense oligonucleotides that modulate aberrant RNA splicing and isoform switching have become available, offering new hope for previously incurable diseases. Here, we review this crucial yet underexplored layer of transcriptomic regulation in human disease, encompassing regulatory mechanisms, technological advances, therapeutic strategies, and future directions.

## Introduction

The human genome catalogs more than 20,000 protein-coding genes, more than 95% of which are multiexonic, with an average of approximately 9 exons per gene (1). Through selective inclusion or exclusion of exons, alternative splicing generates transcript isoforms that directly impact protein sequences and modulate functional elements, including transcript stability, localization, translational efficiency, and the retention or loss of regulatory elements. These processes are tightly coordinated to support physiological demands of distinct tissues, defining and reinforcing cell identity, plasticity, and intercellular interaction. While these diverse attributes grant functional complexity and adaptability at the cellular and organismal level, splicing dysregulation is known to compromise normal cell physiology, carrying serious implications for disease progression. Indeed, altered splicing machinery and mis-spliced isoforms have been implicated in a broad range of human diseases, including neurological and muscular disorders, cardiovascular diseases, autoimmune diseases, fibrotic and metabolic diseases, and cancer (2–5). Moreover, altered peptide sequences may serve as neoantigen substrates, rewiring immune microenvironments, particularly in cancer (6, 7).

Splicing abnormalities were first characterized via gel electrophoresis and Sanger sequencing of cDNA clones. The launch of next-generation sequencers led to an era of high-throughput profiling of splicing events at large scale. More recently, long-read sequencing has emerged as a transformative approach for resolving full-length transcripts across bulk, single-cell, and spatial contexts. With the growing recognition of splicing-related pathogenesis, therapeutic strategies that modulate RNA splicing and isoform switching have become a new class of interventions for previously incurable diseases.

Although the origins, mechanistic basis, regulation, and pathophysiological significance of alternative splicing have been extensively reviewed (8–13), here, we focus on the functional impact of transcript isoforms in human disease, particularly the insights gained from genomics-driven efforts. We examine how technological advances in RNA sequencing have transformed the identification and interpretation of transcript isoforms. Last, we provide an overview of the current state of splicing-based therapeutics.

## Mechanisms of transcript structure diversity

**Figure 1:**
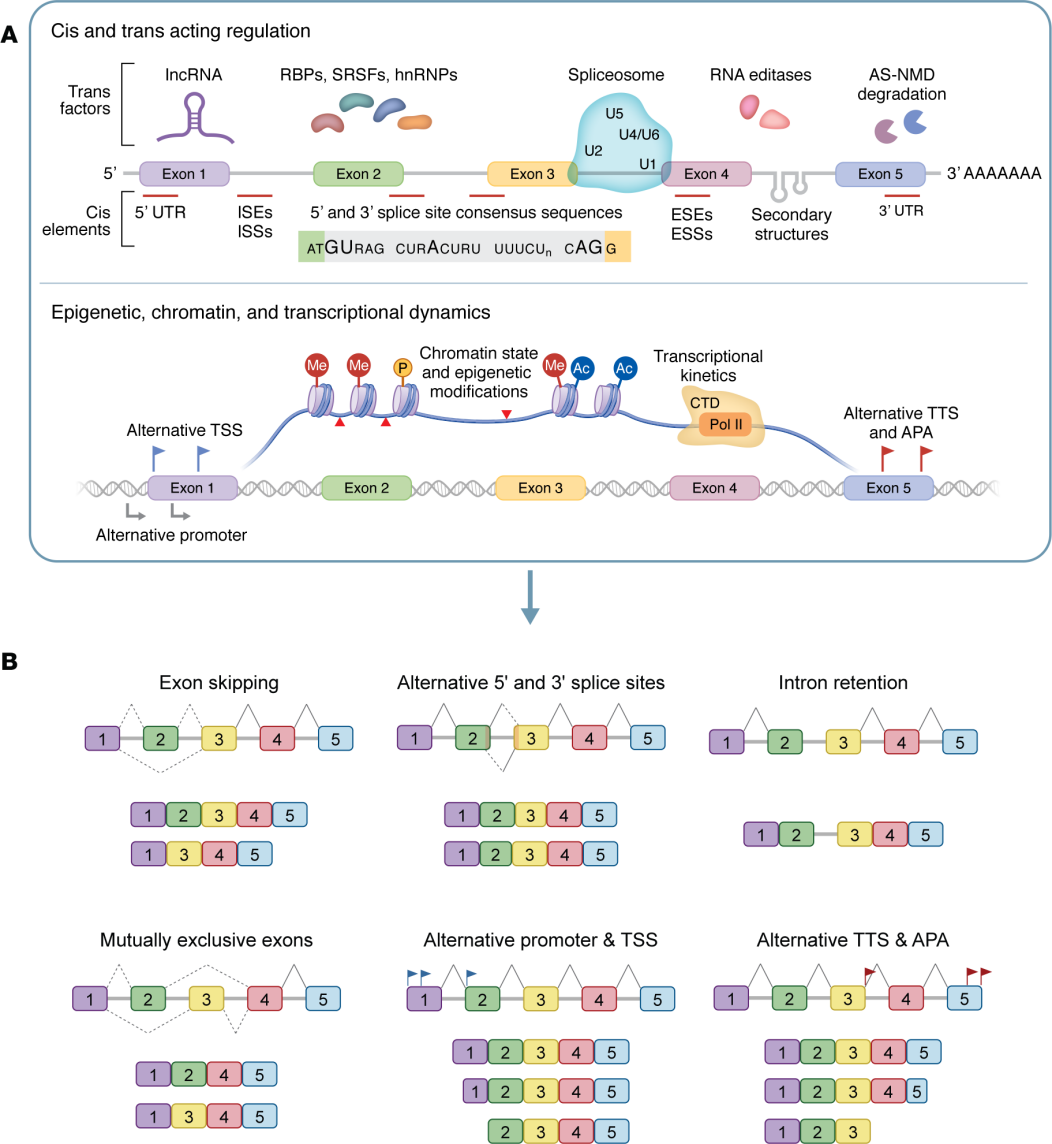
A dense regulatory network dictates isoform diversity. (**A**) *Cis*-regulatory factors encompass the 5′ and 3′ UTRs, exonic and intronic splicing enhancer and silencers (ESEs, ESSs, ISEs, ISSs), the 5′ and 3′ splice site consensus sequences, and RNA secondary structures. Sources of *trans*-splicing regulation include spliceosomal machinery, RNA-binding proteins (RBPs), splicing factors, long noncoding RNAs (lncRNAs), RNA editases, and alternative splicing with nonsense-mediated decay (AS-NMD) coupling. The coordination between *cis*- and *trans*-regulation determines alternative splicing event decisions. Epigenetic, chromatin, and transcriptional regulation encompass alternative promoters, transcription start sites (TSSs), histone modifications, transcriptional elongation rate, alternative transcription termination sites (TTSs), and alternative polyadenylation sites (APAs). (**B**) Splicing events can be categorized as exon skipping, alternative 5′ and 3′ splice sites, intron retention, mutually exclusive exons, alternative promoters, alternative TSSs, and APAs. snRNPs, small nuclear ribonucleoproteins; SRSFs, serine and arginine rich splicing factors; hnRNPs, heterogeneous nuclear ribonucleoproteins; Pol, polymerase.

While alternative splicing serves as the predominant contributor of transcript diversity, nonsplicing sources of diversity, such as alternative promoters, transcription start sites (TSSs), transcription termination sites (TTSs), and polyadenylation, represent an emerging focus in understanding transcriptome complexity (Figure 1). Moreover, recent data highlight the roles of transcriptional rate, chromatin state, and epigenetic modifications in splicing outcomes, suggesting a higher degree of coordination between RNA processing and gene expression than previously thought. The mechanistic and regulatory basis of these processes lays the foundation for the extensive repertoire of transcript isoforms, supporting both broad and specialized functions at the cellular, tissue, and organismal levels.

### Alternative splicing–derived transcript diversity.

Alternative splicing generates transcript isoforms that vary in exon and intron composition from the same gene. The majority of introns (i.e., U2-type introns) are bound by 5′ and 3′ splice sites, which feature the core dinucleotides GU and AG, respectively. These splice sites, together with a core branch point adenosine and polypyrimidine tract located upstream of the 3′ splice site, constitute the essential elements of splicing by the major spliceosome. The spliceosome, composed of the core snRNPs U1, U2, U4, U5, and U6, along with associated splicing factors, assembles onto splice site motifs on pre-mRNA to catalyze two sequential transesterification reactions, producing the lariat intermediate and completing exon ligation (8, 14, 15). Minor introns (i.e., U12-type), accounting for <0.4%–1% of introns in human genes, feature less efficient AU-AC splice site motifs with a distinct branch point sequence, which mediate excision by the minor spliceosome (16, 17). The precise patterns of intron removal and exon ligation largely determine the identity of the mature transcript isoform and can be classified into several distinct alternative splicing events (18).

A common event generating alternative isoforms is cassette exon splicing, or exon skipping, in which an exon is selectively removed from the pre-mRNA. While constitutive exons are essential and almost always included in mature mRNA, cassette exons predominantly function as modular elements that encode tissue-specific protein domains, functional motifs, and flexible regions, providing a major source of protein diversity (19, 20). Cassette exons are often characterized by weaker splice sites, shorter length, and lower GC content compared with constitutive exons (21). A widely studied cassette exon is exon 7 of survival motor neuron 2 (*SMN2*), which produces a nonfunctional protein isoform when skipped (22). As reviewed in detail below, therapeutic interventions in spinal muscular atrophy (SMA) aim to promote *SMN2* exon 7 inclusion to rescue the function of its paralog, *SMN1*, due to homozygous loss.

The spliceosome may also mediate cleavage proximal to canonical splice sites, known as alternative splice sites. Splicing at these alternative sites may shift the 3′ and 5′ boundaries of the upstream and downstream exon, respectively, producing transcript isoforms with altered protein-coding sequences, open reading frames (ORFs), or regulatory motifs (23, 24). The exact cleavage site is determined by regulatory elements embedded in the pre-mRNA and their interaction with splicing factors. Alternative splice sites can also naturally occur within transcript bodies, and their selective recognition by splicing factors contributes to shifts in isoform usage. A representative example is the alternative 5′ splice site selection of exon 2 of the *BCLX* (aka, *BCL2L1*) gene, which produces two splice variants that exert opposing effects on apoptosis in cancer (25).

Intron retention events, also widely observed, occur when an intron that is normally excised during splicing is retained in the mature transcript. These introns often disrupt the frame of the transcript and render it nonproductive. Accordingly, the mechanism is a common mode of regulation that controls transcript abundance in a tissue- and cell type–specific manner, as well as in response to developmental cues, stress, and disease (26–28). Retained introns may also serve as a signal for nuclear retention, affecting turnover and regulating splicing completion (29–31). In some cases, transcripts harboring retained introns maintain an ORF and are translated to produce functional protein isoforms. These unique introns usually encode amino acids within disordered regions and are termed “exitrons” (exonic introns) (32, 33).

A small subset of alternative splicing events involves mutually exclusive exons (MXEs), in which only one exon from a set of two or more is retained in the mature mRNA, while the others are excised. MXEs often encode alternative versions of a protein domain and were previously thought to originate from the same ancestral exon. Large-scale RNA-seq studies characterizing more than 1,300 MXEs have since shown little sequence homology across MXEs, many of which are embedded within secondary structural elements (34). A classic example of MXEs is found in the fibronectin gene *FN1*, which contains 2 MXEs encoding either extra domain A or extra domain B, conferring distinct functions in extracellular matrix remodeling, tissue repair, and cell differentiation (35).

### Non-splicing-derived transcript structure diversity.

A large portion of transcript variability is introduced outside of spliceosome-mediated cleavage and ligation. These differences emerge predominantly at the transcriptional level, encompassing alternative promoters, alternative TSSs, and alternative TTSs. In many cases, alternative promoters produce transcript isoforms that differ in the first exon, resulting in the presence or absence of distinct N-terminal protein domains that contribute specialized functional activities (36–38). In comparison, alternative TSSs represent a finer scale of transcript variation, distinguished by the initiation of transcription at different positions within a common promoter. This mechanism can generate transcript variation that influences posttranscriptional regulation in the form of increased 5′ UTR length, the inclusion of repressive elements, and translationally repressive upstream ORFs (39). Alternative TTSs, also referred to as alternative cleavage and polyadenylation sites, contribute to the production of distinct isoforms by defining heterogeneity at the 3′ UTR, a critical component that regulates RNA subcellular localization and turnover (40, 41). Additionally, variation of the terminal exon may produce key protein isoform derivations, as exemplified by the secreted and membrane-bound forms of the IgM heavy chain in B cells (42). More than half of human genes have been reported to encode two or more canonical polyadenylation signals, with differential usage associated with cell and tissue types, extracellular cues, developmental stage, and disease (43, 44).

## Regulation of transcript isoform diversity

The mechanisms generating transcript diversity are regulated through a coordinated interplay among *cis*-acting elements, the *trans*-acting factors that bind or influence them, and more recently appreciated cotranscriptional, epigenetic, and biophysical dynamics. Further regulatory mechanisms include RNA editing and NMD, as well as splicing regulation mediated by lncRNAs.

### Cis-regulatory elements.

By convention, *cis*-acting elements are motifs embedded within the pre-mRNA, with the consensus sequences of 5′ and 3′ splice sites, branch points, and polypyrimidine tracts representing the archetypal features that regulate splicing activity. These motifs vary in conservation, strongly influencing direct recognition and binding by core spliceosome machinery (45). Less evolutionarily constrained *cis*-acting elements are exonic and intronic splicing enhancers and silencers (ESEs, ESSs, ISEs, ISSs), which are often short motifs that recruit a diverse array of RBPs and splicing factors (13, 46, 47). As mentioned, pre-mRNA may also harbor multiple polyadenylation signals, canonically AAUAAA hexamers flanked by upstream or downstream auxiliary elements, that mediate cleavage and polyadenylation efficiency capable of exerting strong influence on the 3′ UTR (40, 48). While not strictly encoded, RNA secondary structures are a unique class of *cis*-acting elements increasingly recognized as splicing regulators, functioning by sterically masking linear *cis*-acting motifs. These structures (e.g., hairpins, stem-loops, and G-quadruplexes) have also been shown to serve as binding sites for RBPs and play an active role impeding transcription, allowing for dynamic regulation of splicing events (49, 50). The significance of pre-mRNA secondary structures is demonstrated by silent mutations that destabilize a stem-loop proximal to the 5′ splice site of Tau exon 10, leading to increased exon inclusion and pathogenesis of frontotemporal dementia (51).

### Trans-acting regulatory factors.

*Trans*-acting factors traditionally encompass the spliceosome components, splicing factors, and RBPs, which collectively regulate splicing activity by recognizing *cis*-acting motifs and modulating spliceosome activity. The regulatory roles of splicing factors and RBPs largely depend on their expression patterns, which are tissue and cell type dependent (52). Individual splicing factors can recognize a broad array of RNA motifs, further amplifying their complex roles in regulation (53). These properties are largely dictated by RNA-binding motifs, enabling broad classification of splicing factors into families such as serine-arginine–rich (SR) proteins and hnRNPs (54, 55).

Given the programmed expression and broad specificity of splicing factors, recent attention has shifted toward transcriptional dynamics and the chromatin landscape to explain how specific interactions between *cis*- and *trans*-acting factors are achieved in regulating splicing outcomes. In addition, increasing data support the kinetic coupling of alternative splicing and RNA Pol II elongation leading to preference of distinct splicing events, with the inclusion of exon 33 of fibronectin transcripts among the most well-characterized cases (56). The nonrandom enrichment of nucleosomes at exon-intron boundaries also suggests chromatin structure as a regulator of splice site decisions, modulating elongation kinetics and indirectly recruiting splicing factors through epigenetic modifications (57, 58). Emerging models describe the impact and coordination of RNA Pol II elongation kinetics, chromatin structure, and histone modifications on isoform diversity (59, 60).

RNA editases (e.g., ADARs and APOBECs) directly modify RNA sequences, affecting over 60% of human transcripts via RNA-editing events, typically A-to-I or C-to-U conversions on single-stranded or double-stranded RNA (61). In addition to their role in antiviral immunity, these conversions are known to tune *cis*-regulatory elements and, consequently, splice site decisions (62, 63). Furthermore, the expression of many genes encoding RNA editases is tissue restricted, allowing for context-dependent editing tailored to specific tissue and cell conditions (64).

NMD, a posttranscriptional surveillance mechanism, serves as a key pathway that modulates isoform abundance (65, 66). In this process, premature termination codons (PTCs) within exons encountered during translation are recognized by degradation factors (e.g., UPF1), which recruit decay factors that promote decapping and exonucleolytic degradation. Controlled inclusion of PTCs couples alternative splicing with NMD (AS-NMD), generating unproductive isoforms selectively targeted for degradation, thereby autoregulating gene and protein expression under various cellular conditions (67). Indeed, large-scale analyses have predicted that over one-third of alternatively spliced isoforms in humans are prone to NMD (68), with a more recent study establishing a link between aberrant splicing and high production of isoforms that bear a PTC, further underscoring the widespread regulatory role of AS-NMD (69).

The lncRNA class represents another potent regulator of alternative splicing dynamics. Direct interactions with splicing factors modulate their phosphorylation state and nuclear localization, as exemplified by the *NEAT1* and *MALAT1* lncRNAs (70, 71). Alternatively, many lncRNAs form DNA-RNA heteroduplexes (i.e., R-loops) that remodel chromatin, affecting histone modifications and RNA Pol II kinetics, both of which tune splicing decisions (59, 72). Directly influencing splice site selection, lncRNAs may duplex with pre-mRNA, as observed between *SAF* lncRNA and *FAS* pre-mRNA. That interaction promotes exon 6 skipping, producing an isoform that decreases sensitivity toward FasL-induced apoptosis in tumor cells (73).

## Pathological splicing alterations and disease phenotypes

While alternative splicing provides the genome with immense transcriptional versatility, it also introduces vulnerabilities to dysregulation and pathogenic errors. Here, we illustrate well-characterized disorders arising from the mechanisms described above, highlighting the etiological implications of splicing pathogenesis in human diseases.

### Localized cis-acting mutations.

A large portion of disease-associated mutations exert their primary functional impact through the disruption of RNA splicing rather than direct alterations to protein-coding sequences (74). These variants primarily occur within *cis*-regulatory elements, particularly through the destruction of the 5′ or 3′ splice boundaries, leading to exon skipping or intron retention. A more complex class of mutations involves the activation of cryptic splice sites within either introns or the affected exons, creating de novo splice site consensus sequences that disrupt normal splicing regulation. When they occur in introns, these aberrant sites lead to the recognition of noncoding sequences as pseudo-exons. Conversely, when activated within exons, they trigger the use of an alternative, internal boundary, leading to the partial deletion of exonic sequences. Both outcomes typically introduce premature stop codons or internal deletions that compromise protein integrity. In addition, mutations may occur within auxiliary regulatory elements (e.g., ESEs, ISEs, ESSs, ISSs) that bind SR proteins/hnRNPs to regulate pre-mRNA splicing, promoting or inhibiting exon inclusion.

Such splicing defects have been documented across a wide range of human diseases (Table 1). Many mutations linked to Duchenne muscular dystrophy (DMD) target the canonical splice junctions of the dystrophin (*DMD*) gene, leading to exon skipping and disruption of the ORF. This frameshift introduces a premature stop codon, resulting in the loss of functional dystrophin and progressive muscle wasting characteristic of the DMD phenotype (75–77). Another classic example is β-thalassemia, an inherited disease driven by *cis*-acting splicing mutations in the *HBB* gene, which activate cryptic splice sites and eventually cause the loss or reduction of β-globin production (78, 79). The two most common variants involved in β-thalassemia are the IVS1-110G>A and IVS2-654C>T mutations. The IVS1-110G>A variant, prevalent in the Middle East, the Mediterranean, and Cyprus, generates a novel splice acceptor within the first intron of *HBB*, leading to the inclusion of a 19-nucleotide intronic fragment that triggers a premature stop codon (80, 81). The IVS2-654C>T variant, common in East Asian populations, activates a de novo 5′ splice site deep within the second intron, resulting in the recognition of a 73-nucleotide pseudo-exon (82, 83). Similarly, in Hutchinson-Gilford progeria syndrome (HGPS), the common causal factor is a de novo point mutation in exon 11 of the *LMNA* gene (c.1824C>T). Although this synonymous substitution does not alter the encoded amino acid, it activates a cryptic 5′ splice donor site within the exon, leading to a 150-nucleotide internal deletion in the mature mRNA. The resulting progerin protein exhibits a truncated C-terminus devoid of a critical endoproteolytic cleavage site required for proper lamin A maturation, causing the accelerated aging phenotype characteristic of HGPS (84, 85).

Disruption of auxiliary splicing regulatory elements is also frequently implicated in human disease. As mentioned above, homozygous mutation or deletion of *SMN1* cannot be fully compensated for by its paralog, *SMN2*, due to a single nucleotide substitution (c.840C>T) in exon 7. Although this mutation is synonymous, it disrupts an ESE and creates an ESS. This shift reduces SRSF1 binding while recruiting hnRNP A1/A2, ultimately promoting exon 7 skipping. Consequently, the protein isoform lacks an essential C-terminal site necessary for SMN protein stability and self-oligomerization, leading to the loss of crucial motor functions and rapid degradation (22, 86, 87). In frontotemporal dementia with parkinsonism linked to chromosome 17 (FTDP-17), mutations within the *MAPT* (Tau) gene disturb the delicate balance of alternative splicing. Normal brain function depends on a precise 1:1 ratio of Tau isoforms containing either three (3R) or four (4R) microtubule-binding repeats, a balance maintained by the regulated inclusion of exon 10. In the diseased condition, pathogenic mutations in *MAPT* strengthen an ESE or weaken an ESS, leading to increased inclusion of exon 10 and skewing the equilibrium toward the 4R-Tau isoform. Excessive 4R-Tau triggers toxic neurofibrillary tangles and progressive neuronal death, driving severe neurodegenerative phenotypes (51, 88, 89).

In addition, *cis*-acting mutations are widely observed in human cancers (90–94). In many malignancies, mutations create de novo splice sites that alter the function of tumor suppressors or oncogenes. A prominent example is the tumor-suppressive gene *TP53*, in which a variety of point mutations target nucleotides adjacent to splice sites that cause exon skipping, intron retention, or activation of cryptic splice sites, leading to the production of p53 isoforms that lack transcriptional activity (95–98). Dysregulation of auxiliary splicing elements can also drive oncogenic isoform switching, as exemplified by an alteration in fibroblast growth factor receptor 2 (*FGFR2*), in which disruption of intronic regulatory elements promotes the usage shift from isoforms associated with the epithelial (IIIb) to the mesenchymal (IIIc) phenotype, thereby enhancing epithelial-mesenchymal transition, a critical step in tumor metastasis and increased invasiveness (99).

### Systemic trans-acting splicing dysregulations.

In contrast with *cis*-acting mutations, which typically affect individual genes, *trans*-acting dysregulations exert broader, systemic effects through alterations in the proteins that orchestrate spliceosome assembly and splice site selection. These factors include the core snRNP components of the spliceosome and auxiliary splicing factors like SR proteins and hnRNPs. Disruptions in their expression, activity, or sequence can lead to widespread and coordinated splicing alterations across numerous transcripts, driving complex disease phenotypes.

A notable class of *trans*-acting disorders, termed spliceosomopathies, results from mutations in the ubiquitous components of the spliceosome (100). Despite the essentiality of these proteins, their abnormalities often demonstrate tissue-specific outcomes. A prototypical example is retinitis pigmentosa (RP), which is frequently driven by mutations in genes encoding core components of the U4/U6.U5 tri-snRNP complex, including *PRPF8*, *PRPF31*, *PRPF3*, and *SNRNP200* (101, 102). The mis-splicing events arising from the abnormality of these components is associated with the apoptosis of retinal photoreceptor cells, a key pathological feature in RP. In addition to core spliceosomal defects, aberrant expression or mutation of splicing regulatory factors may underlie tumorigenesis of many human cancers. For instance, recurrent somatic mutations in *SF3B1*, *U2AF1*, and *SRSF2* are known to drive a large portion of myelodysplastic syndromes (MDS) and are associated with myeloid leukemias (103, 104). The most prevalent MDS mutation occurs in *SF3B1*, which causes aberrant recognition of alternative 3′ splice sites, typically ~20 nucleotides upstream of the canonical site, leading to the widespread inclusion of cryptic sequences and resulting in widespread mis-splicing of genes involved in hematopoiesis, mitochondrial function, and DNA repair. Similarly, mutations in *U2AF1* and *SRSF2* disrupt sequence recognition at the 3′ splice site and exon definition, leading to transcriptome-wide changes in exon inclusion and splice site choice.

Beyond genomic mutations, dysregulated expression of splicing regulatory genes is also frequently implicated in human malignancy. For example, overexpression of *SRSF1* has been reported in multiple cancers, promoting splicing dysregulation of genes such as *BIN1*, *MNK2*, and *S6K1*. These aberrant isoforms collectively enhance cell proliferation, inhibit apoptosis, and promote malignant transformation (105, 106). Conversely, altered expression of hnRNP family members can repress exon inclusion and contribute to malignant phenotypes through coordinated changes in alternative splicing networks. For instance, high levels of hnRNPA1 and hnRNPA2 bind to the pyruvate kinase (PKM) pre-mRNA to suppress exon 9 inclusion in favor of exon 10. This orchestrated switching to the PKM2 isoform is recognized as a hallmark of the Warburg effect, facilitating the aerobic glycolysis required for rapid tumor growth (107). Together, these cases demonstrate that even in the absence of primary sequence mutations, disruption of splicing factors can collapse healthy alternative splicing networks and drive complex disease phenotypes.

## Technologies characterizing alternative splicing and isoforms

### Pre-NGS era.

**Figure 2:**
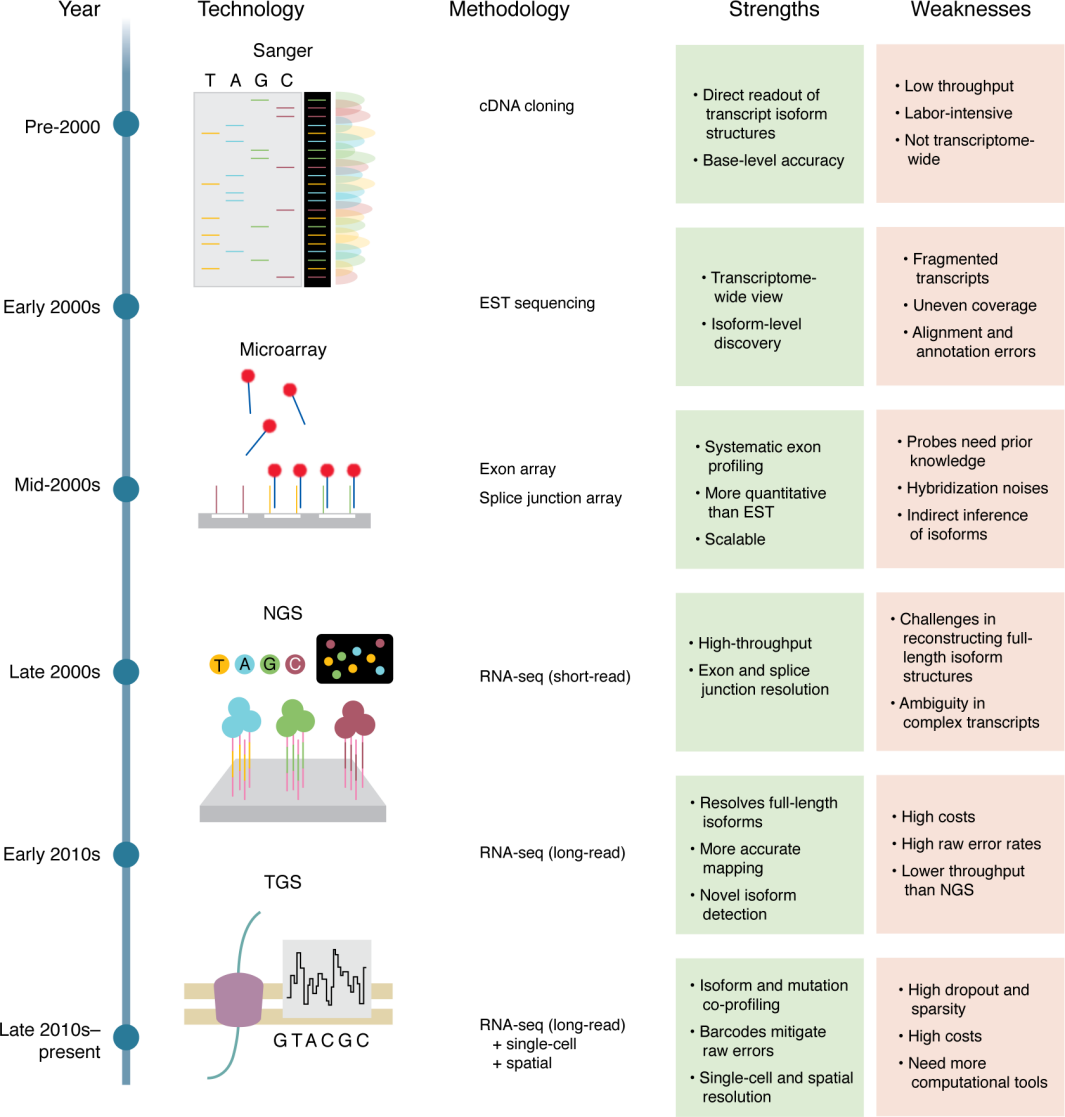
Technological advancements driving isoform-level investigations. Pre-NGS era approaches to study transcript isoforms included cDNA cloning, expressed sequence tag (EST) sequencing, and microarray. These methods offered valuable insights but were generally limited by throughput and high labor demands. NGS technologies introduced high-throughput, transcriptome-wide studies of isoforms but were constrained by read fragmentation, which created ambiguity in full-length transcript abundances. While early platforms exhibited high error rates, accuracy has improved. Tissue and cell resolution can now be achieved when integrated with single-cell and spatial technologies. TGS, third-generation sequencing.

Before the launch of next-generation sequencing (NGS) platforms, the characterization of alternative splicing and transcript isoforms relied primarily on conventional low-throughput molecular techniques (Figure 2). One widely used approach was a cDNA amplification and cloning method that distinguished splicing isoforms based on differences in product sizes (108). Following the establishment of the EST platform (109) by Adams et al., in 1991, advances in full-length cDNA-cloning methods, such as the CAP trapper strategy (110, 111), further drove isoform studies beyond mere gene identification. These developments enabled the systematic discovery of alternative splicing events through EST-to-genome alignment analysis in the late 1990s and early 2000s (112–116). In parallel, pioneering computational frameworks, such as splice graphs by Xing et al. (117), were developed to support genome-wide isoform assembly. Later, microarray hybridization-based exon and splicing arrays provided higher-throughput methodologies for measuring exon-level expression across the genome (118–122). These early approaches established fundamental principles of isoform characterization, yet they were largely limited by low-throughput, incomplete transcript coverage and dependence on prior annotations.

### NGS-based splicing profiling.

The advent of NGS marked a major shift in splicing analysis, enabling large-scale systematic interrogation of transcriptomes. Launched in 2005, the 454 platform was the first successfully commercialized NGS system, which was soon overtaken by Illumina platforms that utilized bridge amplification to generate millions to billions of clonal clusters on a glass flow cell. Due to the inherent constraints of bridge amplification and sequencing-by-synthesis chemistry, Illumina sequencing is limited to short reads, typically ~50–300 bp lengths. Nevertheless, its high yield, accuracy, and throughput enabled deep coverage and precise quantification of splicing events. The deep surveys of alternative splicing in the human transcriptome were reported in 2008 by Pan et al. (123), Sultan et al. (124), and Wang et al. (125), which revealed that approximately 95% of multiexon genes undergo alternative splicing across major human tissues.

As sequencing depth and data scale increased, quantitative characterization of splicing features from large-scale RNA-seq data became challenging. The percent spliced in (PSI or Ψ) metric was first introduced by Wang et al. in 2008 to quantify the ratio of reads supporting the inclusion of an alternative exon to the total number of reads (inclusion + exclusion) (125). As a statistical model, mixture-of-isoforms was applied to estimate the relative isoform dosages and refine PSI values (126). DEXSeq was developed to quantify differential exon usages by testing the changes in exon-level read counts (127). Replicate multivariate analysis of transcript splicing (rMATS), developed by Shen et al. in 2014, introduced replicate-aware models for detecting differential splicing across conditions (128). Along with the development of robust computational methods, large-scale initiatives such as GENCODE (129) and GTEx (130–132) were established to systematically catalog splicing events across human tissues, providing foundational references for modern transcriptome analysis.

### Entering the long-read sequencing era.

While NGS led to advancements in the understanding of alternative splicing and its roles in human disease, studies were limited to analyzing individual splicing sites without informing complete isoform structures. Only a small portion of isoforms could be confidently reconstructed despite advancements in computational methodologies (133–135). These limitations drove the development of long-read RNA-sequencing (LR-RNA-seq) technologies, primarily pioneered by Pacific Biosciences (PacBio) and the Oxford Nanopore Technologies (ONT), which enable direct, isoform-resolved transcriptome profiling.

Introduced in 2011, the first commercialized long-read platform (PacBio RS) employed a single-molecule real-time (SMRT) sequencing technique to enable real-time observation of DNA Pol activity. A key milestone was the development of high-fidelity reads, which drastically improved sequencing accuracy (136). Newer PacBio platforms (Revio and Sequel IIe) have achieved up to 100 gigabase yields per SMRT Cell. Through the Multiplexed Arrays Sequencing (MAS-Seq) workflow, the throughput of transcriptomic isoform sequencing (ISO-Seq) is increased by concatenating individual amplicons of cDNAs into long molecules for sequencing.

In a fundamentally different approach, the ONT nanopore-based sequencing technology determines nucleotide bases by measuring characteristic changes in the ionic current as individual DNA or RNA molecules pass through a protein nanopore under an applied voltage. The first commercial ONT sequencer, MinION, supported ultralong reads exceeding 100 kilobases and the direct sequencing of native RNA molecules (137). The technology has since advanced toward higher throughput and improved accuracy for large-scale sequencing applications. PromethION platforms now achieve ultrahigh yields, ranging from hundreds of gigabases to multi-terabase scales, supporting large-scale full-length transcriptome studies.

Full-length transcript characterization via advanced computational tools, such as IsoQuant (138), Bambu (139), and others (140–150), has enabled the profiling and discovery of thousands of transcript isoforms, both known and novel. To date, a tremendous number of LR-RNA-seq datasets (>300 studies in humans) have been deposited in the NCBI GEO, encompassing full-length isoform assemblies across diverse tissues and conditions (147, 151, 152). However, the vast majority represent bulk approaches that average signals across heterogeneous cell populations, obscuring cell type– and cell state–specific isoform patterns.

### Across single-cell and spatial dimensions.

Emerging long-read single-cell RNA-sequencing (LR-scRNA-seq) technologies barcode full-length cDNAs of single cells and sequence them on high-yield long-read sequencing platforms. To address high error rates in barcode sequences, early LR-scRNA-seq approaches often relied on matched short-read data to facilitate accurate barcode detection and data demultiplexing, as leveraged by ScISOr-Seq in 2018 and ScNaUmi-seq in 2020 (153, 154). In 2023, Shiau et al. presented scNanoRNAseq and an accompanying computational tool, scNanoGPS, which fully removed the dependence on matched short-read data (155), representing a transition to a long-read-only, single-cell workflow. Applying this approach, Pan et al. reported a comprehensive cell-level isoform atlas of the adult human heart and heart failure, revealing hundreds of cell type–specific and disease-associated isoform usage-shifting events (156). Concurrently, Al’Khafaji et al. introduced MAS-ISO-seq, combining PacBio MAS-seq with single-cell barcoding to enable isoform-level quantification in individual cells (157). In parallel with technical advances, analytical approaches have shifted from categorizing splicing events toward more accurate quantification of isoform usage and diversity in cells. Computational frameworks, such as DEXSeq and Hypatia (127, 158), have been adapted or developed to enable comparative analysis of isoform complexity across cell populations. Similarly, long-read spatial transcriptomics have advanced by combining cDNA-based spatial transcriptomic platforms such as Visium (10x Genomics) with long-read sequencing to reveal the spatial mapping of full-length isoforms and splicing regulation (150, 159, 160). Despite these advances, current methods remain limited by dropout, although targeted enrichment strategies partially mitigate data sparsity.

### New insights enabled by emerging technologies.

Recent long-read sequencing studies have led to the identification of previously unappreciated classes of splicing defects, such as multiexon skipping, exonic intron creation, intronic polyadenylation, combinatorial splicing defects, and thousands of novel isoforms that are missed by short reads (161–163). Notably, direct linkages between genetic variants and splice isoforms established from LR-RNA-seq data enabled the diagnosis of multiple rare diseases and revealed new RNA-processing events, such as cryptic intronic polyadenylation activation and haplotype-specific splicing variants (164).

Moreover, emerging single-cell and spatial LR-RNA-seq technologies have further advanced the discovery of isoform usage shifts across cell types, cell states, and spatial niches, providing a foundational framework for understanding previously unrecognized disease mechanisms. In cancer, the identification of hundreds of tumor cell–enriched isoforms (155, 158) offers a rich source of candidate targets for selective tumor cell killing, with the potential to minimize effects on normal tissues. In heart failure, the discovery of cardiomyocyte-specific isoform-switching events (156) may inform more translationally relevant studies and therapeutic strategies. In addition, neoantigens derived from tumor cell–specific, protein-coding isoforms present new opportunities for oncoimmunotherapies. Furthermore, the resolution of cell type– and cell state–specific isoforms with full-length characteristics may enhance the fidelity of in vitro disease models, enabling more accurate recapitulation of cellular phenotypes and fate trajectories.

## Therapeutic strategies targeting alternative splicing and isoforms

**Figure 3:**
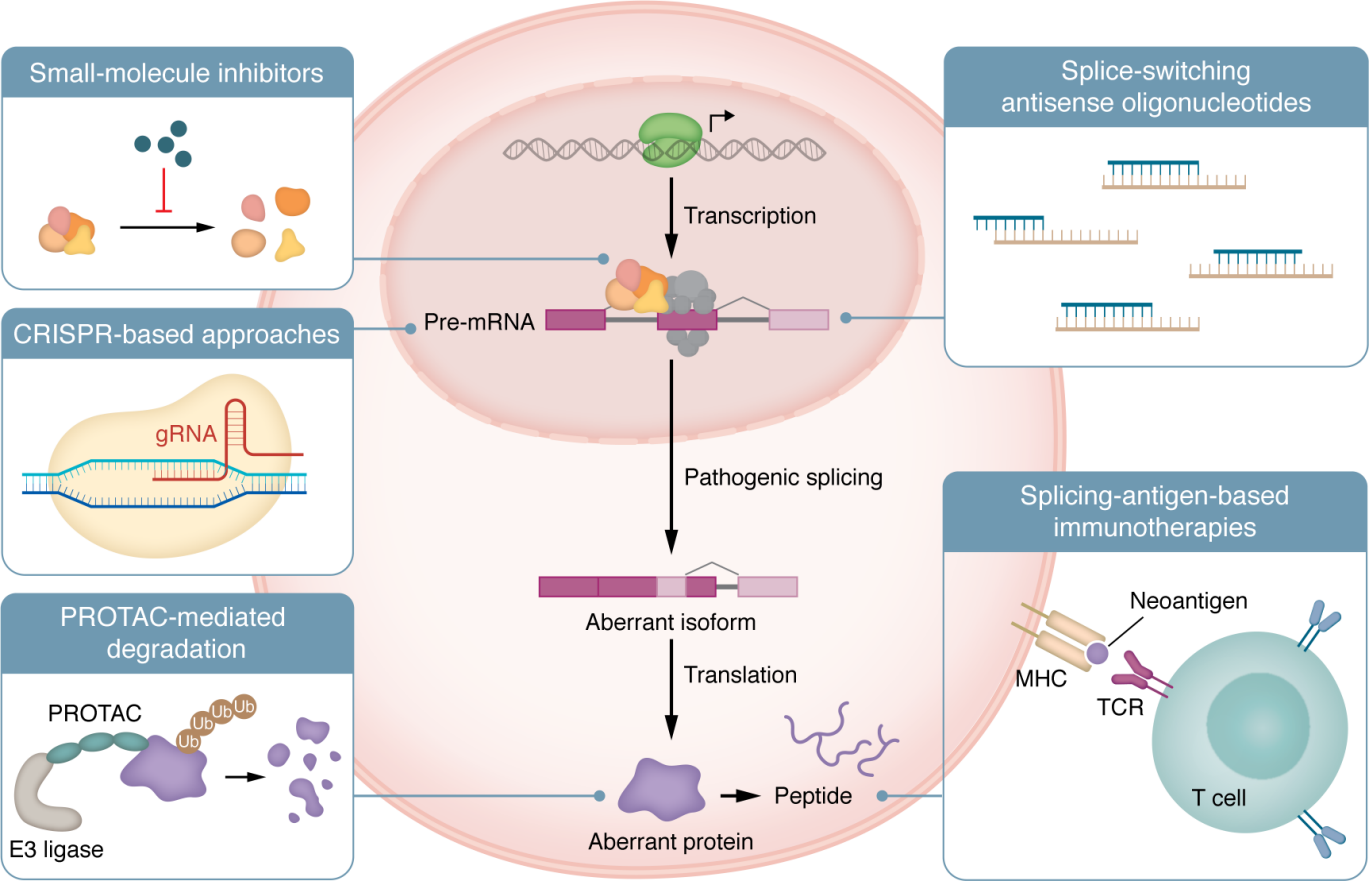
Therapeutic modalities targeting alternative splicing and isoforms in human diseases. Small-molecule inhibitors bind spliceosome and splicing factors, preventing generation of aberrant isoforms. Antisense oligonucleotides (ASOs) base-pair to pre-mRNA, blocking aberrant splicing sites. CRISPR-based approaches target pre-mRNA or DNA to correct/disrupt aberrant splice sites. Splicing-antigen–based immunotherapies involve engineering immune cells to recognize isoform-derived neoantigens. PROTAC-mediated degradation approaches recruit an E3 ligase for ubiquitination and proteasomal degradation of aberrant proteins. PROTAC, proteolysis-targeting chimera.

Growing recognition of the critical roles of alternative splicing and isoforms in human diseases has driven the development of multiple therapeutic strategies (Figure 3). The most widely studied approaches are small-molecule inhibitors and antisense oligonucleotides (ASOs). CRISPR-based approaches have recently expanded rapidly. Splicing-antigen– based immunotherapies and PROTAC-mediated targeted degradation of aberrant isoforms have emerged as novel therapeutics. Clinical-grade modulators of RNA splicing have become available, providing new hope for isoform-driven diseases that were previously considered incurable. Here we focus on the unique isoform-targeting paradigms that have demonstrated therapeutic benefits, highlighting approaches that directly target splicing regulation and isoform usage.

### Small-molecule modifiers.

Early splicing-targeting strategies focused on identifying natural products that inhibit functional components of the spliceosome. In 2008, O’Brien et al. reported the discovery of isoginkgetin, a natural biflavonoid isolated from *Ginkgo biloba* leaves that is membrane permeable and inhibits pre-mRNA splicing by disrupting recruitment of the U4/U5/U6 tri-snRNP to the pre-spliceosome (165). The antitumor drug E7107 is another natural product that can block spliceosome assembly (166). As research continued, more compounds targeting splicing factors and spliceosomes were identified through in vitro splicing assays (165, 167), including H3B-8800, an FDA-approved orally available small-molecule splicing modulator that targets mutated SF3B1 to selectively kill cancer cells (168).

Modulation of pre-mRNA structure offers an additional strategy to selectively target mRNAs by binding to secondary or tertiary structural elements, thereby promoting or inhibiting the inclusion of specific exons in pathogenic genes. In 2020, risdiplam (Evrysdi) became the first FDA-approved, orally administered small-molecule splicing modulator for treating SMA. It functions by targeting a weak secondary structure at the 5′ splice site of the pre-mRNA of *SMN2* to stabilize its interaction with the spliceosome and overcome an inhibitory stem-loop structure. This precise structural stabilization ensures the inclusion of exon 7, allowing for the production of the full-length functional SMN protein essential for motor neuron survival (169).

### ASOs.

Splice-switching antisense oligonucleotides (SSOs) are a widely studied and clinically proven therapeutic strategy for selective targeting of pathogenic splicing and isoforms. SSOs are a distinct class of ASOs (typically 15–30 nucleotides) that complement specific regions of pre-mRNA to interfere with splice site recognition, modulating splicing and preventing the production of pathogenic isoforms. The first generation of naked (unmodified) ASOs were highly unstable in vivo as they were subject to various endo- and exonuclease degradation (170, 171). A foundational optimization was achieved through the introduction of phosphorothioate (PS) modification, which greatly enhanced ASO resistance to RNase H cleavage and in vivo half-life (172, 173). However, the PS modification was frequently associated with off-target interactions and toxicities (174, 175). To ameliorate these effects, the PS backbone modification was combined with 2′ sugar modifications and locked nucleic acid (LNA) chemistry (176). These approaches resulted in higher binding affinity and specificity (177, 178) and are widely adopted in modern SSO drugs.

A successful example of SSOs is nusinersen (Spinraza), approved for SMA, an 18-mer 2′-MOE-PS oligonucleotide that binds to an intronic splicing silencer (ISS-N1) in *SMN2*, forcing the inclusion of exon 7 to produce stable, full-length protein. In cancer, SSOs are applied to rewire *BCL2L1* isoform usage, as the isoform BCL-xL drives antiapoptotic signaling and therapeutic resistance, whereas the isoform BCL-xS promotes apoptosis and treatment sensitivity. SSO-mediated redirection of splicing from BCL-xL to BCL-xS demonstrated robust antitumor effects in multiple studies (179). Further examples include SSO targeting of exon 15 of *HER2* to reduce the full-length transcripts in breast cancer (180). Similarly, LNA-based oligonucleotides targeting androgen receptor expression exhibit antitumor activity in prostate cancer cells (181). However, although the number of genes successfully targeted with PS backbone SSOs is growing, their cellular toxicity and retention remain incompletely resolved.

A safer alternative, charge-neutral phosphorodiamidate morpholinos (PMOs), exhibit reduced nonspecific protein binding and lower risk of hepatotoxicity and immune activation (177). Due to their weak interactions with cell membranes, PMO-based strategies often require high doses to mitigate inefficient cellular uptake and limited tissue penetration, especially in nonmuscle tissues. In contrast with the wide usage of PS-based SSO agents across tissue types, the effective and approved PMOs predominantly target neuromuscular disorders. Eteplirsen (Exondys 51) is approved for DMD, which employs a PMO-based strategy to induce the skipping of mutated exon 51 in the dystrophin gene (*DMD*) and restore the ORF. Casimersen (Amondys 45), another PMO, is approved for treating DMD via exon 45 skipping of *DMD*. Moreover, both golodirsen (Vyondys 53) and viltolarsen (Viltepso) are approved PMOs for exon 53 skipping of *DMD*.

### CRISPR/Cas-based genome editing.

CRISPR/Cas-based technology has recently evolved into a precise tool for transcriptomic engineering by enabling the genomic editing of *cis*-regulatory elements in pre-mRNAs. Unlike SSOs or small-molecule drugs that act transiently at the RNA level, CRISPR/Cas-based strategies allow for durable and potentially permanent correction of aberrant splicing programs in the genome. Although still in active development, they have shown great potential for precise editing of transcript isoforms. Yue et al. successfully rewired the expression of the long- or short-splicing isoforms of the mouse *Xist* gene by modifying the 5′ splice site in intron 7 using the CRISPR/Cas9 system (182). Du et al. reported an engineering system of CRISPR Artificial Splicing Factors (CASFx) that enhanced inclusion of exon 7 of *SMN2* in SMA patient fibroblasts (183). Yuan et al. reported a versatile genetic platform for modulating RNA splicing by using CRISPR-guided cytidine deaminase to convert guanines to adenines at 5′ or 3′ splice sites, thereby correcting the aberrant splicing in disease (184). These early studies reveal considerable promise of applying CRISPR/Cas-based systems for precise isoform corrections yet raise safety concerns because of the off-target effects and permanent genomic alterations.

Recent advances in RNA-targeting and -editing technologies have expanded the capability of the CRISPR-based therapeutic toolkit. CRISPR-Csm (Type III) systems are programmable, RNA-guided, RNA-targeting tools for precise knockdown of nuclear and cytoplasmic transcripts without high off-target cleavage or permanent genome editing (185). Combinatorial RNA Engineering via Scaffold Tagged gRNA (CREST) further extends this approach by enabling simultaneous alternative splicing modulation and RNA base editing through multifunctional transcriptome engineering, while reducing off-target editing by nearly 99% (186). In parallel, CasRx/dCasRx-based platforms have emerged to regulate alternative splicing by recruiting splicing effectors or sterically modulating splice site recognition, allowing for precise, reversible exon-level isoform modulation while avoiding direct genome editing (187). Collectively, these RNA-targeting platforms offer powerful and programmable tools for precisely regulating pathogenic isoforms and address off-target concerns associated with earlier genome-editing methods.

### Targeted protein degradation.

PROTACs offer a transformative strategy to selectively eliminate aberrant splicing factors and protein isoforms, particularly those previously considered undruggable (188). Isoform-specific targeting is possible with ligand-driven PROTACs, in which ligands uniquely bind to the targeted protein isoforms to recruit E3 ligases that confer degradation. Qiu et al. demonstrated that the PROTAC-based degrader SIAIS361034 can selectively degrade the antiapoptotic protein BCL-xL and inhibit tumor growth while having low side platelet toxicity compared with conventional inhibitors (189). Interestingly, Ghidini et al. introduced a new concept of RNA-PROTACs for targeting RBPs by employing small RNA mimics as targeting groups that dock the RNA-binding site of the RBP while conjugating with E3-recruiting peptides for proteasomal degradation (190). Although currently under development and evaluation, PROTAC-based approaches address a key limitation of conventional small-molecule inhibitors by overcoming the challenge of high sequence homology between protein isoforms, enabling improved selectivity.

## Conclusion and future directions

Alternative splicing and isoform switching represent critical mechanisms of gene regulation and protein diversity in humans. Integrating isoform biology with disease mechanisms opens new avenues for precision medicine. Advances in long-read single-cell and spatial transcriptomics are transforming our ability to resolve full-length isoforms across diverse cell populations and spatial contexts, enabling a more comprehensive understanding of transcript diversity. Further integration with genomic and proteomic alterations and functional annotation will enhance the identification of disease-relevant isoforms and therapeutic targets. Translating isoform-level insights into clinically actionable therapeutic strategies remains a key objective. While new classes of therapeutics have rapidly emerged, the development of precise targeting strategies via integrative genomic and proteomic approaches holds promise for more durable treatments of splicing-associated diseases.

## Conflict of interest

The authors have declared that no conflict of interest exists.

## Funding support

This work is the result of NIH funding, in whole or in part, and is subject to the NIH Public Access Policy. Through acceptance of this federal funding, the NIH has been given a right to make the work publicly available in PubMed Central.

- Support provided to RG by the National Institute of General Medical Sciences (NIH R35GM142539) and the National Heart, Lung, and Blood Institute (NIH 1R01HL160552-01).

## Figures and Tables

**Table 1:**
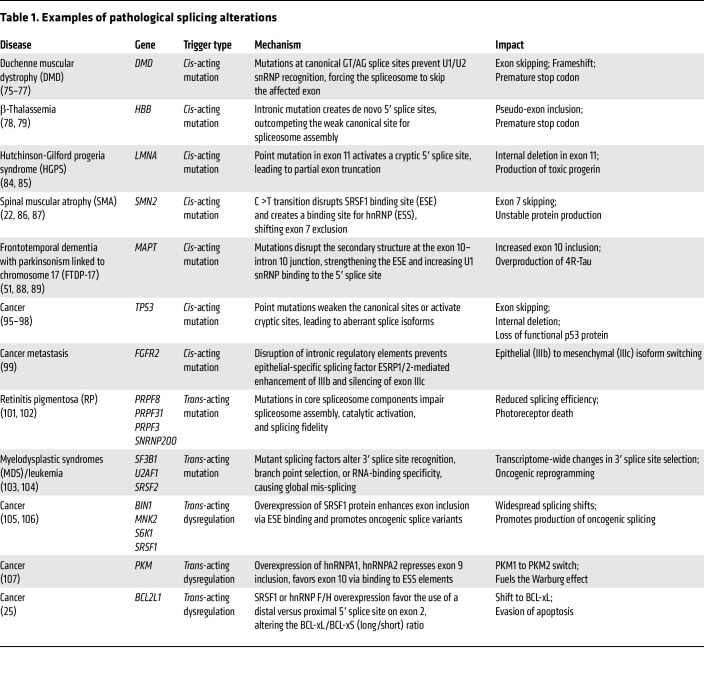
Examples of pathological splicing alterations

## Version 1 07/15/2026

Electronic publication
